# Supplementary material for: Inhibition of acetic acid-induced colitis in rats by new Pediococcus acidilactici strains, vitamin producers recovered from human gut microbiota
Source: PLoS One. 2021 Jul 26;16(7):e0255092. doi: 10.1371/journal.pone.0255092 (PMC8312973; doi:10.1371/journal.pone.0255092)
Supplement: S1 Table — (DOCX) [file pone.0255092.s001.docx]

**S1 Table: The antibiotics susceptibility of the isolates; WNYM01, WNYM02 and WNYM03.**

| **Antibiotic** | **Concentration (ug)** | **Inhibition Zone (mm) for the three isolates** | | |
| --- | --- | --- | --- | --- |
|  |  | **WNYM01** | **WNYM02** | **WNYM03** |
| **Ampicillin** | 10 ug | - (R) | - (R) | - (R) |
| **Amoxicillin** | 30 ug | 15 (I) | 15 (I) | 20 (S) |
| **Erythromycin** | 15 ug | 36 (S) | 29 (S) | 30 (S) |
| **Ciprofloxacin** | 15 ug | 11 (R) | - (R) | - (R) |
| **Gentamycin** | 10 ug | 20 (S) | 20 (S) | 20 (S) |
| **Chloramphenicol** | 30 ug | - (R) | - (R) | - (R) |
| **Vancomycin** | 30 ug | - (R) | - (R) | - (R) |
| **Tetracycline** | 30 ug | 29 (S) | 32 (S) | 30 (S) |
| **Kanamycin** | 25 ug | 15 (I) | 15 (I) | 15 (I) |
| **Co-trimoxazole** | 25 ug | 15 (I) | 15 (I) | 15 (I) |
