# Supplementary material for: Inhibition of acetic acid-induced colitis in rats by new Pediococcus acidilactici strains, vitamin producers recovered from human gut microbiota
Source: PLoS One. 2021 Jul 26;16(7):e0255092. doi: 10.1371/journal.pone.0255092 (PMC8312973; doi:10.1371/journal.pone.0255092)
Supplement: S2 Table — The rat experimental groups: control, received PBS only; Ulcerative, received PBS and colitis induction; A, received WNYM01 + colitis induction; B, received WNYM02 + colitis induction; C, received WNYM03 + colitis induction. M, received Mixture of WNYM (01–03) + colitis induction. Data are presented as mean ± S.D. (n = 3). *Significance compared to Ulcerative. Mean differences are significant (p < 0.05). (DOCX) [file pone.0255092.s002.docx]

**S2 Table**: Assessment of inflammatory markers in the cytoplasmic extracts from homogenate rat's intestinal tissues tumor necrosis factor-α (TNF-α) and intelukin-10 (IL-10)

|  | **TNF-α** | **IL-10** |
| --- | --- | --- |
| **Treatment A** | 160.5±2.5*** | 229.5±14.5*** |
| **Treatment B** | 143.5±3.5*** | 246±9*** |
| **Treatment C** | 146.5±8.5*** | 261±3*** |
| **Treatment M** | 131±6*** | 281.5±6.5*** |
| **Ulcerative** | 407.5±17.5 | 121±4 |
| **Control** | 104.25±11.75*** | 314±11.14*** |

***significance compared to Ulcerative. (Mean differences are significant (p < 0.05)).**
