# Supplementary material for: Inhibition of acetic acid-induced colitis in rats by new Pediococcus acidilactici strains, vitamin producers recovered from human gut microbiota
Source: PLoS One. 2021 Jul 26;16(7):e0255092. doi: 10.1371/journal.pone.0255092 (PMC8312973; doi:10.1371/journal.pone.0255092)
Supplement: S3 Table — The rat experimental groups: control, received PBS only; Ulcerative, received PBS and colitis induction; A, received WNYM01 + colitis induction; B, received WNYM02 + colitis induction; C, received WNYM03 + colitis induction. M, received Mixture of WNYM (01–03) + colitis induction. Data are presented as mean ± S.D. (n = 3). * Significance compared to Ulcerative. Mean differences are significant (p < 0.05). (DOCX) [file pone.0255092.s003.docx]

:

**S3 Table:** Assessment the Levels of lipid peroxidation marker thiobarbituric acid reactive substances (TBARS) and oxidative stress biomarkers glutathione (GSH) in the cytoplasmic extracts from homogenate rat's intestinal tissues

|  | **TBARS** | **GSH** |
| --- | --- | --- |
| **Treatment A** | 2.675±0.075** | 1.1±0.05*** |
| **Treatment B** | 2.525±0.125** | 1.05±0.02*** |
| **Treatment C** | 2.375±0.025** | 0.919±0.024*** |
| **Treatment M** | 2.2±0.05** | 0.814±0.059*** |
| **Ulcerative** | 6.95±0.45 | 1.617±0.033 |
| **Control** | 1.7±0.1** | 0.631±0.043*** |

*** Significance compared to Ulcerative. (Mean differences are significant (p < 0.05).**
