# Supplementary material for: Inhibition of acetic acid-induced colitis in rats by new Pediococcus acidilactici strains, vitamin producers recovered from human gut microbiota
Source: PLoS One. 2021 Jul 26;16(7):e0255092. doi: 10.1371/journal.pone.0255092 (PMC8312973; doi:10.1371/journal.pone.0255092)
Supplement: Attachment — Submitted filename: Dear Editor of PLOS ONE.docx [file pone.0255092.s004.docx]

Dear Editor of PLOS ONE

Please, find the review of the manuscript PONE-D-21-15668 entitled "Inhibition of Acetic acid-Induced Colitis in Rats by P. acidilactici WNYM (01-03); Vitamin Producers Recovered from Human Gut Microbiota"

**Major comments:**

1- Lack of references in many paragraphs inside the manuscript as follows:

- 2^nd^ paragraph in the Introduction section includes many information without references.
- Last line in page 3 (many studies ??) where are the references of these studies.

2- Methods section also there are many references lacking as follows:

- Acid Tolerance assay ??
- Blood hemolysis
- HPLC for determination of riboflavin & folate
- Determination of oxidative stress biomarkers

3- Expression of the results in the form of tables or figures are necessary & more impressive as text only is not enough as follows:

- Screening the isolation by PCR
- Photo of Gel electrophoresis Band.
- Molecular identification
- HPLC peak point & retention time

4- Gross finding: because you administered the bacterial strains for 14 days before colitis induction, So, this is not treatment regimen, it is prophylactic or preventive strategy. If you administer the strain after colitis induction start, at this case become treatment regimen.
5- Discussion section: there is no discussion for the antibacterial activity of the bacterial strains??
6- Also, the results of antioxidant activity of the bacterial strain are not discussed & no interpretation has mentioned.

**Minor corrections:**

a- Abbreviation in the title is not preferred (P. Pediococcus)
b- First sentence in the second paragraph in introduction section to be revised.
c- Introduction: 2^nd^ paragraph: Vitamin B ~~lack~~ (un common word) to be changed in to Vitamin B deficiency; Also respiratory contagion to be changed to respiratory infection.
d- Page (4), second paragraph, second sentence is very long need to be revised as not logic to contain (and & furthermore) in the same sentence.
e- Page (10): first paragraph last line, the word ~~after~~ to change to	"Later".
f- Result section: isolation of LAB: no need to repeat the first 5 lines as it is mentioned in the method section & the results start definitely from the line 6.
